# Peritoneal Lipomatosis: A Case Report of a 12-Year-Old Boy

**DOI:** 10.1155/2013/496419

**Published:** 2013-05-13

**Authors:** L. Fotis, J. Koglmeier, N. Shah

**Affiliations:** ^1^Department of Pediatrics, P. & A. Kyriakou Children's Hospital, Athens, Greece; ^2^Paediatric Gastroenterology Department, Great Ormond Street Hospital for Children, Great Ormond Street, London WC1N 3JH, UK

## Abstract

Peritoneal lipomatosis is a rare disease in childhood with only two cases previously described in children. We report a further case of a 12-year-old boy diagnosed with peritoneal lipomatosis. His main symptoms were abdominal pain, alternating bowel habit, abdominal distension, and melaena. His diagnostic work up included an abdominal MRI, wireless capsule endoscopy and single-balloon enteroscopy. Peritoneal lipomatosis although rare can be diagnosed in childhood. It is a benign clinical entity with variable manifestations.

## 1. Introduction

Lipomatosis of the peritoneum is a rare disorder with less than 50 cases published to date [1]. There are only two previously reported cases in childhood. We describe a further case of a 12-year-old boy diagnosed with peritoneal lipomatosis. We report the findings of MRI and endoscopic assessment by wireless capsule endoscopy (WCE) and Single-balloon Enteroscopy (SBE).

## 2. Case Report

**Figure 1:**
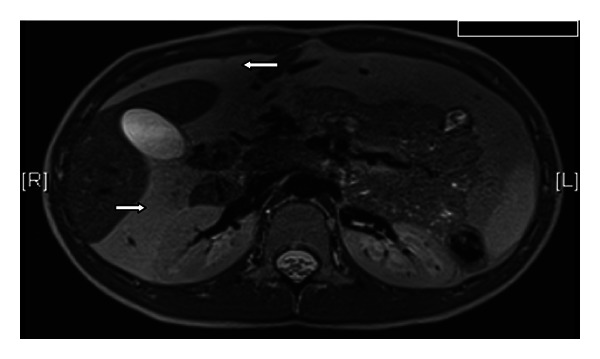
MRI abdomen: lipomatous tissue anterior to the liver and between the right lobe of the liver and the right kidney.

**Figure 2:**
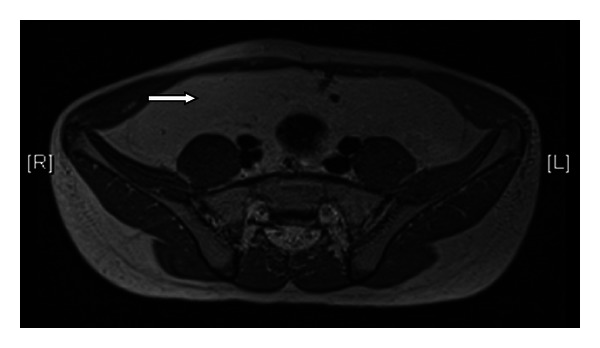
MRI pelvis: lipomatous tissue in the pelvis.

**Figure 3:**
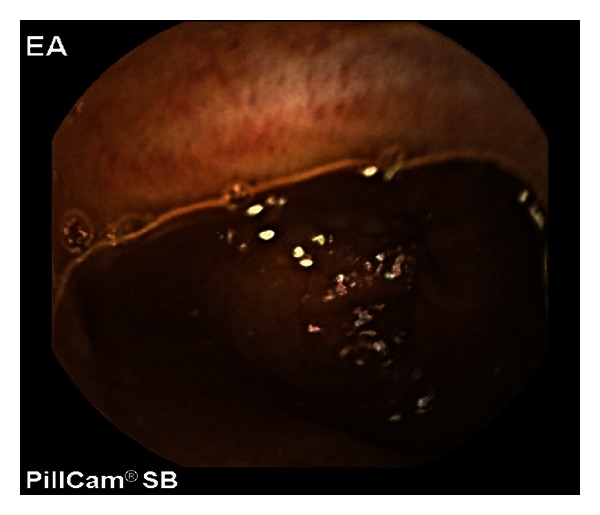
Wireless capsule endoscopy: yellow, round, and conceited area of the small bowel.

A two-year-old boy initially presented to his local hospital prominent gastrocolic reflex, bloating, and intermittent changing shape of his abdomen. At the age of 4.5 years he had an abdominal U/S due to ongoing symptoms, which was suggestive of peritoneal lipomatosis. As he continued to complain about abdominal pain, urgency and alternating bowel habit he was referred for further evaluation to our specialist centre at 12 years of age. At the time other symptoms were noted, including intermittent abdominal distention and occasional blood in the stools. Radiological imaging was initially done by MRI scan which revealed widespread peritoneal lipomatosis, encasing the intraperitoneal contents with the lipomatous tissue lying anterior to the liver and stomach. It extended between the right lobe of liver and right kidney and down to the peritoneal cavity into the pelvis. The lipomatosis displaced the bowel loops centrally. The liver spleen, kidneys, and pancreas appeared normal (Figures 1 and 2). The WCE (Pillcam SB, Given Imaging, Yoqneam, Israel) did not reveal any significant abnormalities apart from ill-defined yellowish, round, lesions (Figure 3). As the boys symptoms did not settle he underwent a SBE (Olympus, Tokyo, Japan). Multiple mucosal biopsies were taken which showed normal mucosa from the duodenum to the distal jejunum/proximal ileum. No specific therapy was given, and his symptoms improved spontaneously except for very mild nonspecific abdominal pain. 

## 3. Discussion

Lipomatosis is a distinct clinicopathologic entity characterized. By the development of nonencapsulated lipomas in subcutaneous tissues [2]. Lipomas are well-defined, noninvasive, benign, and encapsulated tumours with a composition similar to that of normal adipose tissue [3]. In generalised lipomatosis, there are masses of diffuse infiltrating lipomatosis resembling simple lipomas except for their extensive infiltrative distribution [3]. Involvement of the face, neck, extremities, trunk, abdomen, and the pelvis has been reported [2]. 

Pelvic, abdominal, and intestinal lipomatosis are distinct clinical entities. They are characterised by abdominal distension as a consequence of intraperitoneal and retroperitoneal fat and respiratory distress due to mediastinal airway compression [2]. Pelvic lipomatosis may present with bladder dysfunction, constipation, nonspecific abdominal discomfort, oedema of the lower extremities, and ureteral obstruction leading to hydronephrosis and renal failure [4]. So far two cases of abdominal lipomatosis have been reported in children. The first case described a five-year old boy who presented with periumbilical nonradiating abdominal pain, abdominal distension and umbilical herniation [3]. The second case was an eight-year old child diagnosed with diffuse lipomatosis including intraperitoneal, retroperitoneal, and abdominal wall involvement [5]. To the best of our knowledge this is the third reported case in the literature of peritoneal lipomatosis with the most extensive series of investigations to evaluate this condition so far.

Our patient is the first reported case of peritoneal lipomatosis investigated by panenteroscopy using WCE and enteroscopy. The patient suffered from altered stool habit, occasional blood in stools, pain, and abdominal distension. Despite panendoscopic examination and pangastrointestinal tract biopsy assessment, no mucosal pathology could be identified, and despite extensive radiological and blood work up his symptoms could not be explained. Therefore, by the elimination of all other causes, we attributed the symptoms to direct mass effects of the lipomatosis. Peritoneal lipomatosis would be responsible for the mechanical symptoms of distension, causing obstruction intermittently and altering bowel transit time. Nevertheless, this would not explain the symptom of blood, albeit a very occasional symptom at best reported by the family.

This we believe is the first WCE description of this condition with the addition of the rarely performed SBE that examined the entire small bowel of this child along with histological specimens excluding infiltration of the intestinal wall by the lesions.

Our case should not be misinterpreted with intestinal lipomatosis, which is the presence of numerous circumscribed lipomas of the intestine [6]. Less than 50 cases have been described worldwide in the literature with a wide span of presenting ages varying between 20 and 88 years [1]. There is only one reported case of a child. The patient was a 10 year old girl who presented with multiple jejunal and ileal lipomas. Her only presenting symptom was abdominal pain and the diagnosis was made with a computed tomography of the abdomen [7].
